# Probiotics (VSL#3) Prevent Endothelial Dysfunction in Rats with Portal Hypertension: Role of the Angiotensin System

**DOI:** 10.1371/journal.pone.0097458

**Published:** 2014-05-15

**Authors:** Sherzad K. Rashid, Noureddine Idris Khodja, Cyril Auger, Mahmoud Alhosin, Nelly Boehm, Monique Oswald-Mammosser, Valérie B. Schini-Kerth

**Affiliations:** 1 UMR CNRS 7213, Laboratoire de Biophotonique et de Pharmacologie, Faculty of Pharmacy, University of Strasbourg, Illkirch, France; 2 Institut d'Histologie et INSERM U1119, Fédération de Médecine Translationnelle de Strasbourg, Faculty of Medicine, University of Strasbourg, Strasbourg, France; 3 Service de Physiologie et d'Explorations Fonctionnelles, Pôle de Pathologie Thoracique, Hôpitaux Universitaires de Strasbourg, Faculty of Medicine, University of Strasbourg, Strasbourg, France; Facultad de Medicina, Universidad Autonoma Madrid, Spain

## Abstract

**Aims:**

Portal hypertension characterized by generalized vasodilatation with endothelial dysfunction affecting nitric oxide (NO) and endothelium-dependent hyperpolarization (EDH) has been suggested to involve bacterial translocation and/or the angiotensin system. The possibility that ingestion of probiotics prevents endothelial dysfunction in rats following common bile duct ligation (CBDL) was evaluated.

**Methods:**

Rats received either control drinking water or the probiotic VSL#3 solution (50 billion bacteria.kg body wt^−1^.day^−1^) for 7 weeks. After 3 weeks, rats underwent surgery with either resection of the common bile duct or sham surgery. The reactivity of mesenteric artery rings was assessed in organ chambers, expression of proteins by immunofluorescence and Western blot analysis, oxidative stress using dihydroethidium, and plasma pro-inflammatory cytokine levels by flow cytometry.

**Results:**

Both NO- and EDH-mediated relaxations to acetylcholine were reduced in the CBDL group compared to the sham group, and associated with a reduced expression of Cx37, Cx40, Cx43, IK_Ca_ and SK_Ca_ and an increased expression of endothelial NO synthase (eNOS). In aortic sections, increased expression of NADPH oxidase subunits, angiotensin converting enzyme, AT1 receptors and angiotensin II, and formation of ROS and peroxynitrite were observed. VSL#3 prevented the deleterious effect of CBDL on EDH-mediated relaxations, vascular expression of connexins, IK_Ca_, SK_Ca_ and eNOS, oxidative stress, and the angiotensin system. VSL#3 prevented the CBDL-induced increased plasma TNF-α, IL-1α and MCP-1 levels.

**Conclusions:**

These findings indicate that VSL#3 ingestion prevents endothelial dysfunction in the mesenteric artery of CBDL rats, and this effect is associated with an improved vascular oxidative stress most likely by reducing bacterial translocation and the local angiotensin system.

## Introduction

Advanced chronic liver diseases are characterized by generalized progressive vasodilatation, related to portal hypertension (PH), which is especially observed in the splanchnic and pulmonary beds. In a rat model of partial portal vein ligation, Albrades et al. [1] reported that the initial event in response to PH is an up-regulation of vascular endothelial growth factor and endothelial nitric oxide synthase (eNOS) in the intestinal microcirculation. Although animal models have led to a number of hypotheses, the exact mechanism involved in the occurrence of the generalized vasodilatation and hyperdynamic syndrome is incompletely understood [2]–[5]. Several lines of evidence support a role for an increased formation of nitric oxide (NO) in the general vasodilatation in chronic liver diseases in patients [6] and also in the chronic bile duct ligated (CBDL) rat model [2], [4]. In addition, chronic liver diseases in patients and also in experimental PH are characterized by an increased vascular formation of reactive oxygen species (ROS) [7], [8] and the activation of the renal renin-angiotensin-aldosterone system and the local angiotensin system [9]–[11]. Moreover, it has been suggested that bacterial translocation (BT) is an important step in the occurrence of the general vasodilatation and especially in the pulmonary bed [3], [12]. In cirrhosis, BT is defined as the migration of bacteria and/or their products from the intestinal lumen to mesenteric lymph nodes [13] as a consequence of intestinal bacterial overgrowth, impaired host defenses, and/or disruption of the gut mucosal barrier [14], [15]. In cirrhotic rats, BT has been reported to lead to endotoxaemia, which stimulates the expression of TNF-α leading to an enhanced formation of the potent vasodilators NO and carbon monoxide [3]. Thus, reducing BT to improve the excessive oxidative stress and/or to reduce the increased vasodilator factors is an interesting target to improve the general vascular dysfunction in PH. Earlier studies have reported that treatment with either antiobiotics or pentoxifylline, an inhibitor of TNF-α, to prevent BT and/or its consequences improves the vasodilatation in CBDL rats [12], [16] and also in patients with liver cirrhosis [17], [18]. Due to the poor tolerance of pentoxifylline [18] and the increased risk of bacterial resistance with antibiotics, probiotics such as VSL#3 which are well tolerated [19], [20] may be of interest. Thus, the aim of the present study was to evaluate the effect of the probiotic VSL#3 formulation on the vascular dysfunction in an animal model of biliary cirrhosis with PH with a special attention to oxidative stress and the local angiotensin system.

## Materials and Methods

### Ethics statement

This study conforms to the Guide of Care and the Use of laboratory Animals published by the US National Institutes of Health (NIH publication No. 85–23, revised 1996). The present protocol was approved by the local Ethics Committee (Comité Régional d'Ethique en Matière d'Expérimentation Animale, approval AL/01/09/09/05).

### Animal model

Male Wistar rats (10–12 week-old) were anaesthetized with an intraperitoneal mixture of ketamine (80 mg.kg body wt^−1^) and xylazine (4 mg.kg body wt^−1^). Biliary cirrhosis was induced by CBDL. After laparotomy, the bile duct was isolated, double ligated and resected between the two ligatures. Sham rats had laparotomy, underwent mobilization of the common bile duct without ligation. After surgery, rats were housed in a thermo-neutral environment, on a 12∶12 h photoperiod and were provided food and drinking water *at libitum*. Eight CBDL rats and seven sham rats were treated three weeks before and 4 weeks following surgery with the probiotic formulation VSL#3 (VSL Pharmaceuticals, Inc, Towson, MD, USA; *Streptococcus thermophilus, Bifidobacterium longum, Bifidobacterium breve, Bifidobacterium infantis, Lactobacillus acidophilus, Lactobacillus plantarum, Lactobacillus casei, Lactobacillus bulgaricus*) at a dose of 50 billion bacteria.kg body wt^−1^ daily, given in the drinking water. Seven CBDL rats and five sham rats receiving vehicle served as controls.

Four weeks after surgery, rats were sacrificed. The liver and spleen were removed and weighted. Liver samples (taken from the median lobe) from each rat were incubated in the Bouin-Holland fixative and subsequently embedded in paraffin for histological analysis. Segments of the main superior mesenteric artery and thoracic aorta were cleaned, embedded in Tissu-Tek O.C.T. compound (Sakura Finetek France SAS, Villeneuve d'Ascq, France) and snap-frozen for immunofluorescence studies and the determination of the formation of ROS. Samples of blood were taken by heart puncture, and, thereafter, plasma was stored at -80°C for subsequent serological analysis.

### Assessment of liver cirrhosis and portal hypertension

Sections of liver samples (5 µm thick) were stained with hematoxylin, eosin and Masson's trichrome stain and evaluated by light microscopy. Histologically, liver of chronic bile duct ligation showed evidence of intrahepatic tubular duct hypertrophy with severe fibrosis. Portal hypertension is reflected by an increase in the spleen weight.

### Vascular reactivity studies

Vascular reactivity studies on the main superior mesenteric artery were performed as described previously [11]. Briefly, the main superior mesenteric artery was cleaned of connective tissue, cut into rings (2–3 mm in length) and suspended in organ baths containing oxygenated Krebs bicarbonate solution for the determination of changes in isometric tension. After equilibration and functional tests, rings were contracted with phenylephrine (PE, 1 µM) before construction of concentration-response curves in response to acetylcholine (ACh), sodium nitroprusside or levcromakalim. In some experiments, rings were exposed to an inhibitor for 30 min before contraction with PE. Relaxations were expressed as percentage of the contraction induced by PE.

### Immunofluorescence studies

Frozen arteries were cryosectioned at 14 µm. Sections were air-dried for 15 min and stored at −80°C until use. Sections were first fixed with paraformaldehyde at 4%, washed and treated with 10% milk or 5% goat serum in PBS containing 0.1% Triton X-100 for 1 h at room temperature to block non-specific binding. Mesenteric artery sections were then incubated overnight at 4°C with an antibody directed against either eNOS (1/100), small and intermediate conductance calcium-activated potassium channels (SK_Ca_, IK_Ca_, 1/200), or connexins (Cx37, Cx40 and Cx43; 1/100 to 1/200). Aortic sections were incubated with an antibody directed against either angiotensin II (1/500), AT1 receptors (1/400), angiotensin-converting enzyme (ACE, 1/200), nitrotyrosine (1/200), p47phox (1/200), p22phox (1/200) or cyclooxygenase-1 or -2 (COX-1, COX-2, 1/200). Sections were then washed with PBS, incubated with the secondary antibody (1/300, Alexa 488- or 637-conjugated goat anti-rabbit IgG) for 2 h at room temperature in the dark before being washed with PBS and mounted in Dako fluorescence mounting medium (Dako France SAS, Les Ulis, France) and cover-slipped. For negative controls, primary antibodies were omitted.

### Western blot analysis

Mesenteric artery and aortic segments were homogenized in extraction buffer (composition in mM: Tris/HCl 20 (pH 7.5; Q Biogene), NaCl 150, Na_3_VO_4_ 1, sodium pyrophosphate 10, NaF 20, okadaic acid 0.01 (Sigma), a tablet of protease inhibitor (Complete Roche) and 1% Triton X-100 (Euromedex)). Total proteins (10 µg) were separated on SDS-polyacrylamide gels and transferred electrophoretically onto polyvinylidine difluoride membranes (Amersham). Membranes were blocked with blocking buffer containing 5% bovine serum albumin, Tris-buffered saline solution (Biorad) and 0.1% Tween 20 (Sigma) (TBS-T) for 1 h. Membranes were then incubated overnight at 4°C with an antibody directed against either eNOS (1/10000), SK_Ca_ (1/200), AT1 receptors (1/1000), p47phox (1/1000), p22phox (1/1000) or COX-2, (1/1000). Thereafter, membranes were incubated with an appropriate horseradish peroxidase-conjugated secondary antibody and signals were detected using enhanced chemiluminescence (Amersham).

### Determination of vascular and mitochondrial ROS Formation

*In situ* formation of ROS was performed using the method previously described [21]. The redox-sensitive fluorescent dye dihydroethidium (DHE, 2.5 µM) was applied onto 25 µm unfixed cryosections of aorta for 30 min at 37°C in a light-protected humidified chamber. To determine the sources of ROS, sections were incubated with either apocynin (NADPH oxidase inhibitor and antioxidant, 300 µM), L-NA (NO synthase inhibitor, 300 µM), sulfaphenazol (cytochrome P450 inhibitor, 100 µM), indomethacin (cyclooxygenase inhibitor, 10 µM) or inhibitors of the mitochondrial respiration chain (myxothiazol, 0.5 µM+rotenone, 1 µM+KCN, 1 µM) for 30 min at 37°C before the addition of dihydroethidium. The *in situ* formation of mitochondrial ROS was performed using MitoSox (Life Technologies SAS, Saint Aubin, France), a mitochondrion-specific dihydroethidium-derivative fluorescent dye. Briefly, 14 µm unfixed cryosections of aorta were incubated with MitoSox (5 µM at 37°C for 60 min) in a light-protected humidified chamber. Sections were then washed three times, mounted in DAKO and cover-slipped.

### Image analysis

All samples from immunofluorescence and *in situ* ROS formation studies were observed using a confocal laser-scanning microscope (Leica SP2 UV DM IRBE). Quantification of fluorescence levels was performed using FIJI GPL v2 software (http://fiji.sc/Fiji).

### Determination of plasma inflammatory cytokine levels

The determination of the plasma level of TNF-α, IL-1α, MCP-1 and IL-4 was performed using a commercial rat cytokine Kit Flowcytomix (eBioscience SAS, Paris, France), multiple analyte detection kit, according to the protocol supplied by the manufacturer. The raw data were analysed using “The FlowCytomixPro software”.

### Statistical analysis

Values are expressed as means ± SEM. Statistical analysis was performed using either Student's *t* test or an analysis of variance (ANOVA) followed by the Bonferroni post-hoc test as appropriate using GraphPad Prism (version 5 for Microsoft windows, GraphPad software, Inc, San Diego, CA, USA). A *P* value less than 0.05 was considered to be statistically significant.

## Results

### Characteristics and histologic findings

As shown in Table 1, four weeks after surgery, CBDL rats had a significantly increased spleen weight, and an enhanced bile duct proliferation as assessed by histological analysis of liver sections. Chronic intake of VSL#3 for seven weeks starting three weeks before the surgery reduced slightly but significantly the CBDL-induced increase in spleen weight whereas the liver weight was unaffected. The VSL#3 treatment alone had no effect on liver and spleen weight.

**Table 1:** Effect of VSL#3 ingestion on body, liver and spleen weight in both sham and CBDL rats.

	Initial body weight (*g*)	Final body weight (*g*)	Liver (*% total weight*)	Spleen (*% total weight*)
Sham	365±19.5	505±30.2	3.24±0.06	0.20±0.02
Sham+VSL#3	368±14.3	506±26.4	3.33±0.18	0.23±0.02
CBDL	369±16.3	485±30.5	6.69±0.63^*^	0.69±0.05^*^
CBDL+VSL#3	365±15.7	507±47.6	6.02±0.55	0.56±0.09^#^

Values are shown as mean ± SEM of 6 rats. ^*^ *P*<0.05 CBDL vs sham, and ^#^ *P*<0.05 for CBDL+VSL#3 vs CBDL.

### VSL#3 treatment prevents the CBDL-induced impaired EDH but not NO component of the relaxation to acetylcholine in mesenteric artery rings

**Figure 1:**
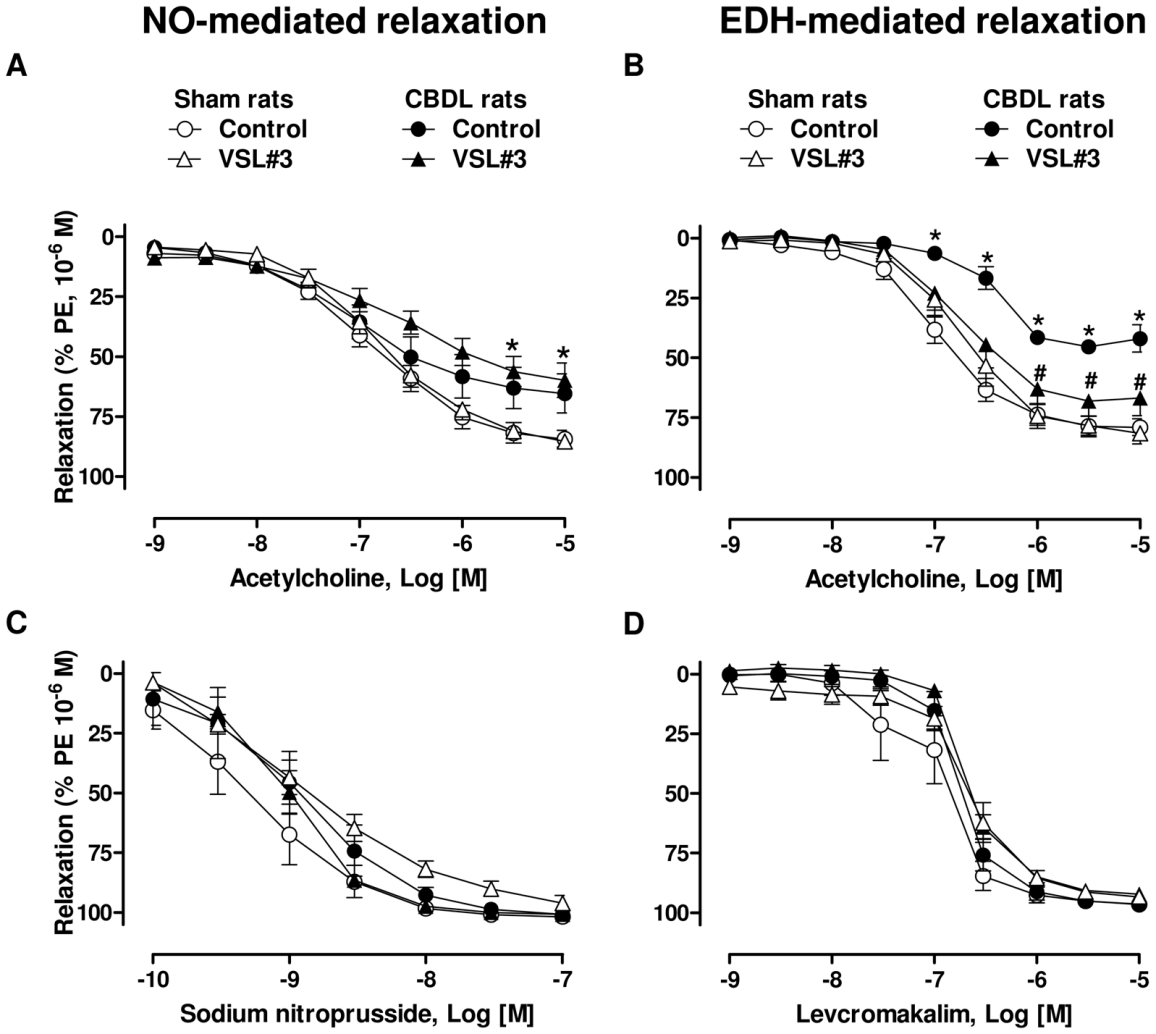
Ingestion of VSL#3 improves the CBDL-induced blunted EDH but not NO-mediated relaxations in mesenteric artery rings. Concentration-relaxation curves to acetylcholine in mesenteric artery rings with endothelium in sham, CBDL, sham+VSL#3 and CBDL+VSL#3 rats. A) The NO component of the relaxation was assessed in the presence of indomethacin (10 µM) and apamin plus charybdotoxin (100 nM each), and B) the EDH component in the presence of indomethacin and N^G^-nitro-L-arginine (300 µM). C) Relaxations to sodium nitroprusside (an exogenous donor of NO) and D) levcromakalim (an ATP-sensitive K^+^ channel opener) in mesenteric artery rings without endothelium are also shown. Results are shown as mean±SEM of 5-7 different rats; *P<0.05 CBDL vs sham, and ^#^ *P*<0.05 CBDL+VSL#3 vs CBDL.

ACh caused similar concentration-dependent relaxations in mesenteric artery rings from control and CBDL rats (maximal relaxations amounted to 97.4±1.4% and 94.9±2.4%, respectively, n = 6–8). However, the NO component of the relaxation as assessed in the presence of indomethacin and charybdotoxin plus apamin to prevent the formation of vasoactive prostanoids and EDH, respectively, was significantly impaired in the CBDL group (Figure 1A). In addition, the EDH-mediated component of the relaxation as assessed in the presence of indomethacin plus N^G^-nitro-L-arginine (an inhibitor of eNOS), was also significantly reduced in the CBDL group (Figure 1B). Although the VSL#3 treatment did not affect the CBDL-induced impaired NO component, it significantly improved the EDH component (Figures 1A and B). In addition, both the sodium nitroprusside (an NO donor) and the levcromakalim (an ATP-sensitive K channel opener) induced endothelium-independent relaxations were similar in all groups (Figures 1C and D).

### VSL#3 treatment prevents CBDL-induced up-regulation of eNOS and down-regulation of SK_Ca_, IK_Ca_, Cx37, Cx40, and Cx43 in the mesenteric artery

**Figure 2:**
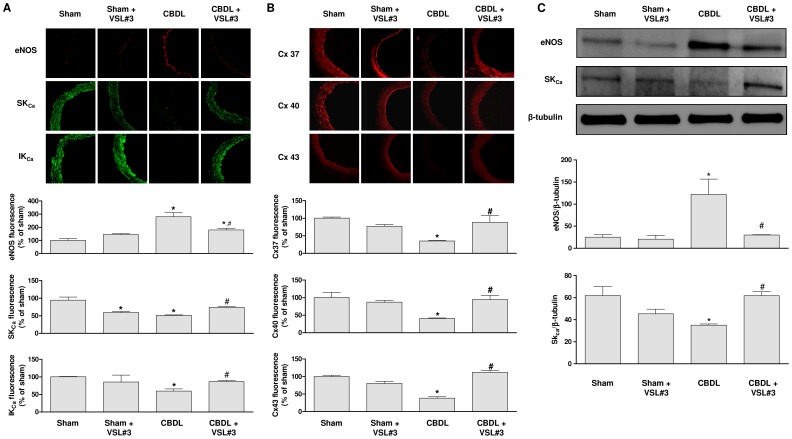
The VSL#3 treatment prevents the CBDL-induced A) up-regulation eNOS and down-regulation of SK_ca_ and IK_ca_, and B) down-regulation of connexins (Cx37, Cx40 and Cx43) in the mesenteric artery. Upper panels show representative immunofluorescence staining, and lower panels corresponding cumulative data. C) Expression of eNOS and SK_Ca_ protein levels as assessed by Western blot analysis. Results are shown as mean±SEM of 4 different rats for A and B, and 3 for C. *P<0.05 for CBDL vs sham, and ^#^ *P*<0.05 CBDL+VSL#3 vs CBDL.

Immunofluorescence studies indicated that the eNOS signal is observed predominantly at the luminal surface of mesenteric artery sections in the control group, and that this signal is significantly increased in the CBDL group (Figure 2A). Pronounced fluorescence signals for SK_Ca_ and IK_Ca,_ two calcium-activated potassium channels involved in EDH [11], are observed throughout the arterial wall in the control group whereas both signals are significantly reduced in the CBDL group (Figure 2A). In addition, the fluorescence signal of connexins Cx37, Cx40 and Cx43, which form myoendothelial gap junctions transmitting the hyperpolarization from endothelial cells to the underlying smooth muscle cells to cause relaxation [11], is observed predominantly at the luminal surface and also to some extent in the vascular smooth muscle (Figure 2B). The fluorescence signals of Cx37, Cx40 and Cx43 are significantly reduced in the CBDL group (Figure 2B). The VSL#3 treatment prevented partially but significantly the CBDL-induced up-regulation of eNOS, and the down-regulation of SK_Ca_ and IK_Ca_, and of Cx37, Cx40 and Cx43 (Figure 2). The VSL#3 treatment of sham rats was without effect except for SK_Ca_, which was significantly reduced (Figure 2). An increased eNOS protein level and a decreased SK_Ca_ level were also observed in the CBDL group as assessed by Western blot analysis (Figure 2C). These effects were significantly prevented by the VSL#3 treatment (Figure 2C). In addition, IK_Ca_ and Cx37 protein levels were below the detection level in all groups.

### VSL#3 treatment reduces the CBDL-induced vascular oxidative stress involving several sources in the aorta

**Figure 3:**
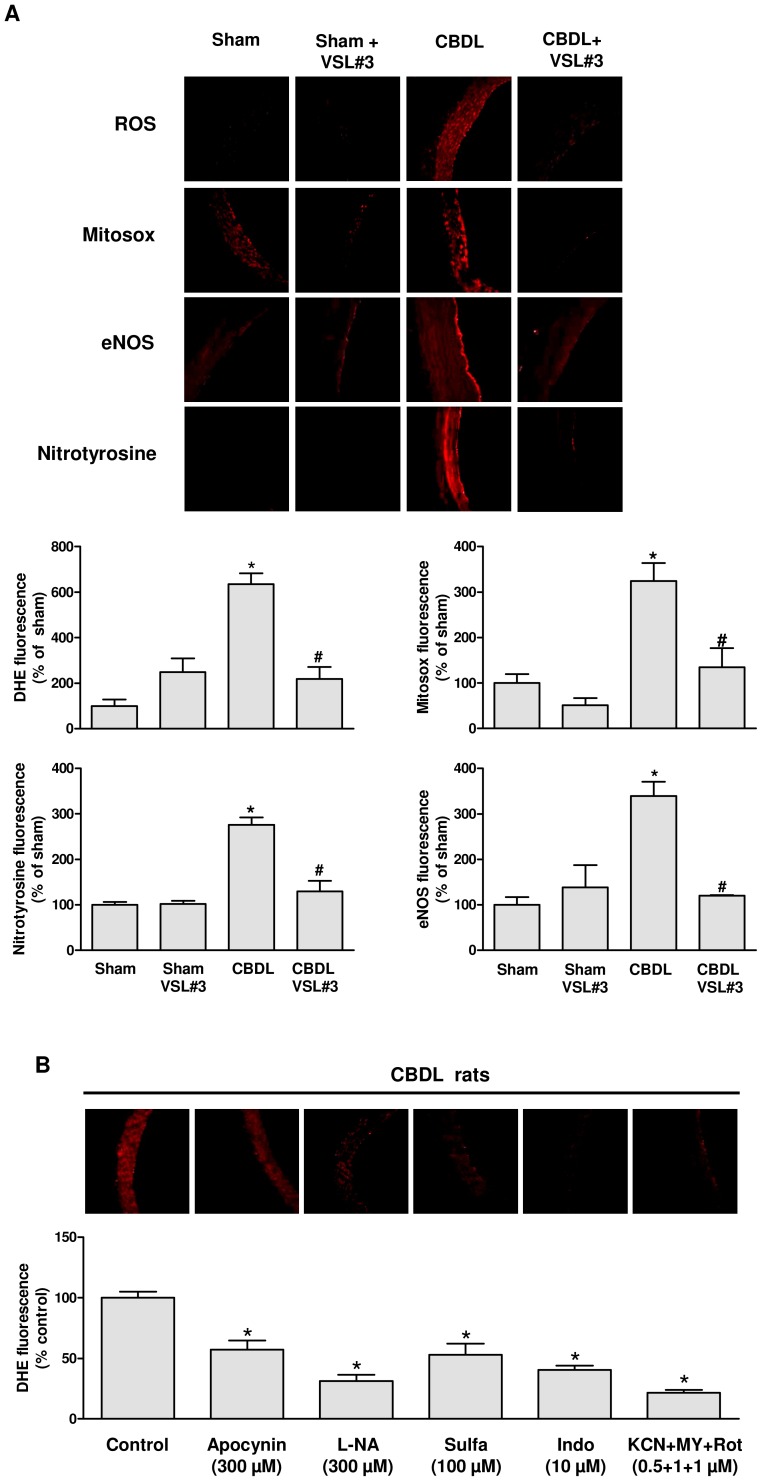
The VSL#3 treatment prevents the CBDL-induced oxidative stress in the aorta. (A) *In situ* determination of the formation of ROS, peroxynitrite (nitrotyrosine), and eNOS in aortic sections. Top: representative immunofluorescence staining; bottom: corresponding cumulative data for 4 to 5 rats/group. (B) To study the cellular sources of ROS, aortic sections were exposed either to apocynin (NADPH oxidase inhibitor and antioxidant), L-NA (NO synthase inhibitor), sulfaphenazol (Sulfa, cytochrome P450 inhibitor), indomethacin (Indo, cyclooxygenase inhibitor) or the combination KCN, myxothiazol and rotenone (KCN+MY+Rot, inhibitors of the mitochondrial respiration chain) for 30 min before DHE staining. Top of each panel: DHE staining; bottom: corresponding cumulative data for 4 rats. Results are shown as mean±SEM. The lumen is on the right side of each image. A) *P<0.05 CBDL vs sham, and ^#^ *P*<0.05 CBDL+VSL#3 vs CBDL; B) *P<0.05 versus control.

**Figure 4:**
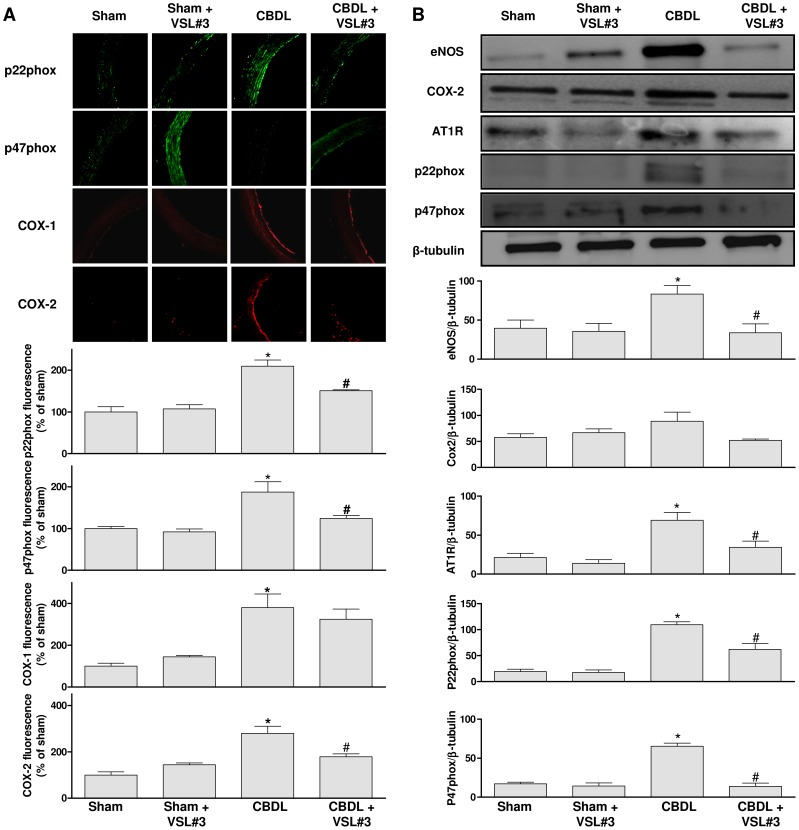
The VSL#3 treatment prevents the CBDL-induced up-regulation of the NAPDH oxidase subunits p22phox and p47phox, and COX-2 but not COX-1 in aortic sections. A) Representative immunofluorescence staining and corresponding cumulative data. B) Expression levels of target proteins as assessed by Western blot analysis in aortic segments. Results are shown as mean±SEM of 4 different rats. *P<0.05 CBDL vs sham, and ^#^ *P*<0.05 CBDL+VSL# 3 vs CBDL.

CBDL markedly increased the DHE fluorescence signal, the MitoSOX fluorescence signal and the nitrotyrosine fluorescence signal throughout the aortic wall, and the eNOS fluorescence signal at the luminal surface and eNOS protein level as assessed by Western blot analysis (Figures 3A and 4B). All these effects were significantly prevented by the ingestion of VSL#3 (Figures 3A and 4B). The VSL#3 treatment of sham rats affected only minimally these signals (Figures 3A and 4B).

The characterization of the cellular sources of oxidative stress has indicated that the CBDL-induced increased DHE fluorescence signal was markedly reduced by apocynin (an NADPH oxidase inhibitor and antioxidant), L-NA (an eNOS inhibitor), sulfaphenazol (a cytochrome P450 inhibitor), indomethacin (a cyclooxygenase inhibitor) and by a combination of inhibitors of the mitochondrial respiration chain (KCN, myxothiazol and rotenone) suggesting the involvement of NADPH oxidase, COXs, uncoupled eNOS, cytochrome P450, and the mitochondrial respiration chain (Figure 3C).

To obtain further evidence for a role of NADPH oxidase and COXs, their expression level was determined by immunofluorescence staining and Western blot analysis in the aorta. A significantly increased immunofluorescence signal of the NAPDH oxidase subunits p22phox and p47phox, and of COX-1 and COX-2 was observed in the aorta of the CBDL group compared to the sham group (Figure 4A). The VSL#3 treatment significantly reduced the CBDL-induced stimulatory effect for p22phox, p47phox and COX-2 whereas that for COX-1 was minimally affected (Figure 4A). The VSL#3 treatment of sham rats affected minimally all these signals (Figure 4A). In addition, an upregulation of p22phox and p47phox protein levels was also observed in the CBDL group as assessed by Western blot analysis whereas no such effect was observed in the CBDL+VSL#3 group (Figure 4B). In contrast, COX-2 protein levels were similar in all groups, and COX-1 protein levels were below the detection level (Figure 4B).

### VSL#3 treatment improves the CBDL-induced expression of the local angiotensin system

**Figure 5:**
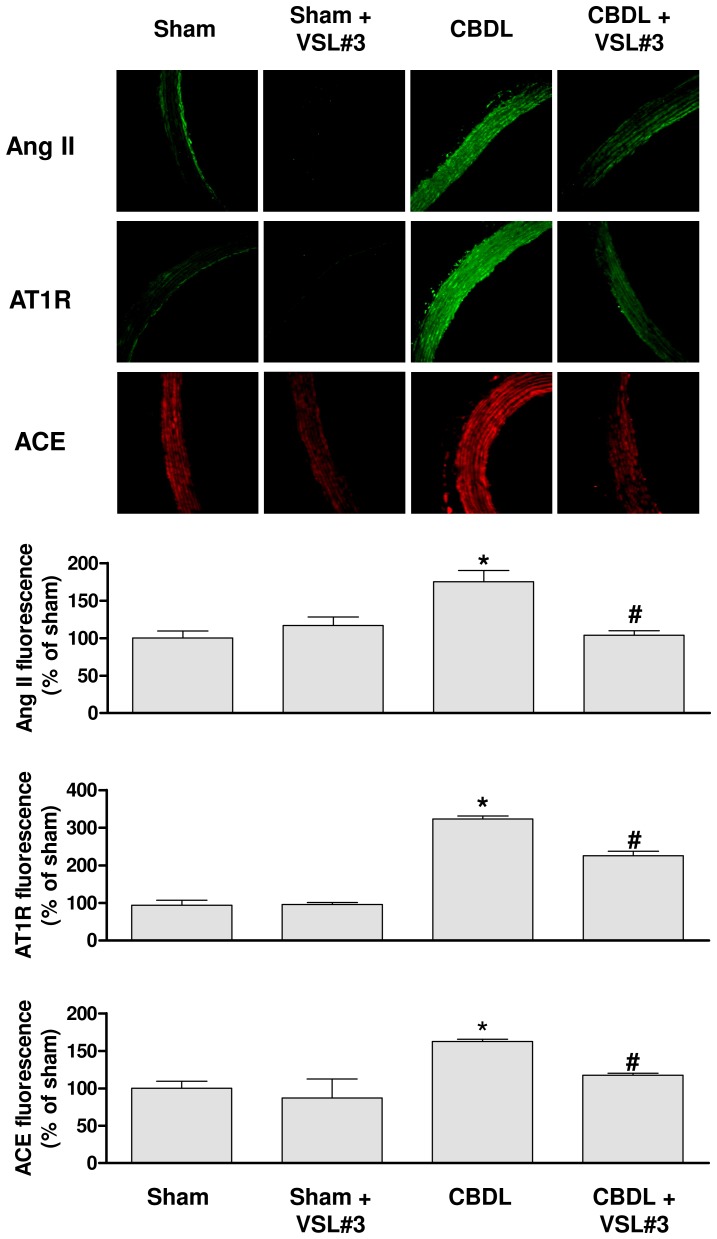
The VSL#3 treatment prevents the CBDL-induced up-regulation of several components of the local angiotensin system in the aorta. The expression level of angiotensin II (Ang II), AT1 receptors (AT1R) and angiotensin-converting enzyme (ACE) was determined by immunofluorescence staining. Upper panels show representative immunofluorescence staining; Lower panels corresponding cumulative data. Results are shown as mean±SEM of 4 different rats. *P<0.05 CBDL vs sham, and ^#^ *P*<0.05 CBDL+VSL#3 vs CBDL.

An increased immunofluorescence level of angiotensin II, AT1 receptors and ACE throughout the aortic wall was observed in the CBDL group (Figure 5). The stimulatory effect of CBDL was significantly prevented by the VSL#3 treatment (Figure 5). The VSL#3 treatment alone affected minimally all these signals (Figure 5). An upregulation of the AT1 receptor protein level was also observed in the CBDL group by Western blot analysis whereas no such effect was observed in the CBDL+VSL#3 group (Figure 4B).

### VSL#3 treatment prevents the CBDL-induced increase in plasma pro-inflammatory cytokines

**Figure 6:**
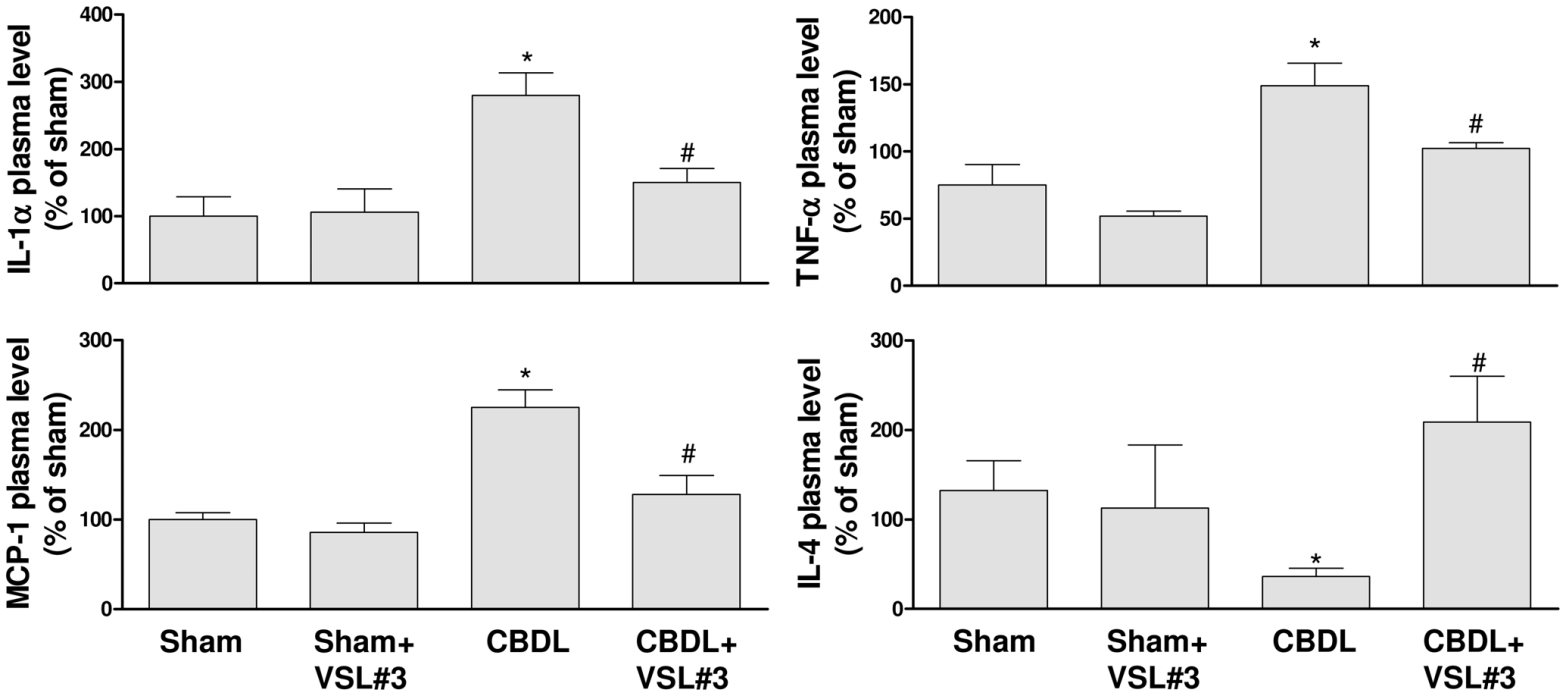
The VSL#3 treatment prevents the CBDL-induced increased formation of pro-inflammatory cytokines including IL-1α, MCP-1 and TNF-α, and decreased formation of the anti-inflammatory cytokine IL-4 in plasma. Results are shown as mean±SEM of 6 different rats. *P<0.05 CBDL vs sham, and ^#^ *P*<0.05 CBDL+VSL#3 vs CBDL.

A significant increase in the plasma level of pro-inflammatory cytokines including IL-1α, MCP-1 and TNF-α was observed in the CBDL group, and this effect was significantly prevented by the VSL#3 treatment (Figure 6). In contrast, the plasma level of IL-4, which is a potent anti-inflammatory cytokine, was markedly reduced in the CBDL group, and this effect was significantly prevented by the VSL#3 treatment (Figure 6). The VSL#3 treatment of sham rats affected minimally all these cytokines (Figure 6).

## Discussion

The present study indicates that the CBDL-induced endothelial dysfunction involves predominantly a reduced EDH component and, also, to some extent a decreased NO component in the mesenteric artery. The blunted EDH component is associated with a reduced expression of SK_Ca_ and IK_Ca_ levels, and Cx30, Cx37 and Cx40 levels, which are all involved in the EDH response, most likely as a consequence of the activation of the local angiotensin system leading to an increased vascular oxidative stress, and the formation of pro-inflammatory mediators. They further indicate that ingestion of the probiotic VSL#3 formulation effectively prevented the CBDL-induced endothelial dysfunction, at least in part, by targeting the vascular angiotensin system.

Biliary cirrhosis induced by chonic bile duct ligation in rats and evidenced by an increased spleen weight and enhanced bile duct proliferation is associated with an unaffected acetylcholine-induced endothelium-dependent relaxation in mesenteric artery rings. In contrast, when experiments were performed to study selectively either the NO component or the EDH component then a significant reduction of both components was observed in the CBDL group as observed previously in some but not all studies [11],[22],[23]. A severely reduced EDH component despite an unaffected ACh-induced relaxation has also been observed previously in aging-related endothelial dysfunction [24]. Such a difference is most likely explained by the fact that the NO component and the EDH component, although blunted, act in synergy to maintain a normal endothelium-dependent relaxation whereas such a compensating mechanism cannot take place when a single component is evaluated. Alternatively, the present findings due not rule out the possibility that vasoactive prostanoids contribute to preserve the acetylcholine-induced endothelium-dependent relaxation in the CBDL group. However, previous findings have indicated that although prostacyclin evoked relaxations in mesenteric resistance arteries from control rats, the prostanoid induced also contractile responses in arteries from spontaneously hypertensive rats presenting an endothelial dysfunction [25]. Moreover, chronic COX inhibition did not affect the CBDL-induced decrease in mean arterial pressure in CBDL rats [26].

The impaired EDH-mediated relaxation is most likely the consequence of the decreased expression in IK_Ca_ and SK_Ca_ involved in EDH formation, and also of Cx37, Cx40 and Cx43, which form myoendothelial gap junctions transmitting the endothelial hyperpolarization towards the underlying smooth muscle [11]. In addition to the present findings, oxidative stress has been suggested to contribute to the impaired EDH-mediated relaxation [11]. The present findings further extent these previous observations by showing that oxidative stress is generated by several sources including NADPH oxidase, uncoupled eNOS, cytochome P450, COXs and the mitochondrial respiration chain. Moreover, an upregulation of several pro-oxidant enzymes including NADPH oxidase, COX-1, and COX-2 is observed in the arterial wall of CBDL rats. Besides the EDH component, the NO component of the relaxation is also impaired in CBDL rats despite an increased expression of eNOS. This latter response is most likely part of a compensatory mechanism due to the degradation of the excessive formation of NO by superoxide anions as indicated by the increased peroxynitrite level.

The present findings support a key role for the angiotensin system in the CBDL-induced endothelial dysfunction. Indeed, an increased expression of ACE, Ang II and AT1R was observed throughout the arterial wall of CBDL rats. They are also consistent with the fact that losartan, an AT1 receptor antagonist, prevented the CBDL-induced endothelial dysfunction and oxidative stress [11]. A major novel finding of the present study is that ingestion of the probiotic VSL#3 formulation improved the endothelial dysfunction in CBDL rats along with a pronounced improvement of the local angiotensin system and the level of oxidative stress in the arterial wall.

Previous studies have shown that amongst factors involved in the general vasodilatation in cirrhosis is bacterial translocation [3], [5], [12]. Indeed, an alteration of the gut microflora associated with disruption of the gut mucosal barrier leading to bacterial translocation has been observed in 45–75% of animals with experimental cirrhosis [14], [15]. This response, in turn, promotes the subsequent inflammatory response, which will contribute to the hyperdynamic circulatory state in cirrhosis [5], [12], [16]. Indeed, treatments preventing bacterial translocation using either antiobiotics or pentoxifylline (an inhibitor of TNF- α) reduced the excessive vasodilatation in CBDL rats [5], [12], [16]. However, the long-term use of antibiotics and pentoxifylline is hazardous due to the risk of inducing bacterial resistance with antibiotics and the poor tolerance of pentoxifylline as reported in a pilot study by Tanikella et al. [18]. In contrast, chronic use of probiotics has not been associated with major side effects [19], [20]. Furthermore, previous studies have indicated that intake of probiotics such as the VSL#3 formulation leads to major changes in the composition of the gut flora in both experimental animals and in humans. Indeed, intake of VSL#3 in patients with pouchitis was associated with an increased intestinal bacterial diversity and a reduced fungal diversity in comparison with patients treated with a placebo [27]. VSL#3 treatment also prevented the antibiotic-induced decrease in several indigenous bacterial groups as assessed using a standardized human fecal microbiota in a computer-controlled model of large intestine [28]. Moreover, intake of the VSL#3 formulation in rats altered the species richness and diversity of the luminal intestinal microbiota [29]. However, the possibility that the beneficial effect of the VSL#3 treatment on the CBDL-induced endothelial dysfunction involves an improved bacterial translocation still remains to be determined.

The study of Loguercio et al. [20] indicated that administration of VSL#3 to alcoholic liver cirrhosis patients led to an improvement of the liver function and the increased plasma level of oxidative stress and TNF-α. In the present study, a relatively small but significant improvement of spleen weights was observed suggesting that VSL#3 may also have some effects on cirrhosis in CBDL rats, which, however, cannot account solely for the improved endothelial function.

In addition in a rat model of cirrhosis induced by CCl_4,_ Chiva et al. [30] reported that the addition of *Lactobacillus johnsonii* to an antioxidant treatment (vitamin C plus glutamate) reduced endotoxemia possibly due to an increased clearance of endotoxins by the monocyte-macrophage system. The VSL#3 treatment also reduced the CBDL-induced pro-inflammatory response as indicated by the improvement of circulating levels of pro-inflammatory cytokines such as IL-1α and TNF-α. Thus, the protective effect of VSL#3 in CBDL rats may involve, besides an improvement of the gut flora composition, possibly also the stimulation of immunological defence mechanisms and perhaps also the recovery of the intestinal motility, which has been shown to be decreased in cirrhosis with bacterial translocation [31].

Another potential protective mechanism of VSL#3 is that upon fermentation, various probiotics (including those contained in VSL#3, [32]) are capable of releasing ACE inhibitory peptides. This has been observed *in vitro* [32], [33] and also *in vivo* in hypertensive rats [34] and patients [35]. In an *in vivo* trial, the ingestion of milk fermented with probiotics led to a significant decrease in blood pressure [35]. In the present study, a normalized vascular expression level of ACE, angiotensin II and AT1R along with a reduced expression of NADPH oxidase was observed in CBDL rats treated with VSL#3. Since infusion of angiotensin II leads to an increased vascular expression of NADPH oxidase and oxidative stress [36], it is likely that the VSL#3 treatment may improve oxidative stress by reducing the local angiotensin system subsequent to the normalization of ACE expression. Alternatively, VSL#3 may also normalize ACE activity via the release of ACE inhibitory peptides as suggested by previous studies [32], [35].

In conclusion, the present findings indicate that oxidative stress possibly due to bacterial translocation inducing a pro-inflammatory response and the local angiotensin system is involved in the endothelial dysfunction in CBDL rats, and that this effect is improved by the ingestion of the probiotic VSL#3 formulation.

## References

[1] Abraldes JG, Iwakiri Y, Loureiro-Silva M, Haq O, Sessa WC, et al (2006) Mild increases in portal pressure upregulate vascular endothelial growth factor and endothelial nitric oxide synthase in the intestinal microcirculatory bed, leading to a hyperdynamic state. Am J Physiol Gastrointest Liver Physiol 290: G980–987. 16603731 10.1152/ajpgi.00336.2005

[2] Ling Y, Zhang J, Luo B, Song D, Liu L, et al (2004) The role of endothelin-1 and the endothelin B receptor in the pathogenesis of hepatopulmonary syndrome in the rat. Hepatology 39: 1593–1602. 15185300 10.1002/hep.20244

[3] Zhang HY, Han de W, Su AR, Zhang LT, Zhao ZF, et al (2007) Intestinal endotoxemia plays a central role in development of hepatopulmonary syndrome in a cirrhotic rat model induced by multiple pathogenic factors. World J Gastroenterol 13: 6385–6395. 18081228 10.3748/wjg.v13.i47.6385 PMC4205458

[4] Zhang J, Ling Y, Luo B, Tang L, Ryter SW, et al (2003) Analysis of pulmonary heme oxygenase-1 and nitric oxide synthase alterations in experimental hepatopulmonary syndrome. Gastroenterology 125: 1441–1451. 14598260 10.1016/j.gastro.2003.07.005

[5] Sztrymf B, Rabiller A, Nunes H, Savale L, Lebrec D, et al (2004) Prevention of hepatopulmonary syndrome and hyperdynamic state by pentoxifylline in cirrhotic rats. Eur Respir J 23: 752–758. 15176692 10.1183/09031936.04.00080404

[6] Gomez FP, Barbera JA, Roca J, Burgos F, Gistau C, et al (2006) Effects of nebulized N(G)-nitro-L-arginine methyl ester in patients with hepatopulmonary syndrome. Hepatology 43: 1084–1091. 16628648 10.1002/hep.21141

[7] Fernando B, Marley R, Holt S, Anand R, Harry D, et al (1998) N-acetylcysteine prevents development of the hyperdynamic circulation in the portal hypertensive rat. Hepatology 28: 689–694. 9731560 10.1002/hep.510280314

[8] Chen MF, Mo LR, Lin RC, Kuo JY, Chang KK, et al (1997) Increase of resting levels of superoxide anion in the whole blood of patients with decompensated liver cirrhosis. Free Radic Biol Med 23: 672–679. 9215813 10.1016/s0891-5849(97)00057-9

[9] Yang YY, Lin HC, Huang YT, Lee TY, Hou MC, et al (2002) Effect of 1-week losartan administration on bile duct-ligated cirrhotic rats with portal hypertension. J Hepatol 36: 600–606. 11983442 10.1016/s0168-8278(02)00037-5

[10] Helmy A, Newby DE, Jalan R, Johnston NR, Hayes PC, et al (2003) Nitric oxide mediates the reduced vasoconstrictor response to angiotensin II in patients with preascitic cirrhosis. J Hepatol 38: 44–50. 12480559 10.1016/s0168-8278(02)00319-7

[11] Dal-Ros S, Oswald-Mammosser M, Pestrikova T, Schott C, Boehm N, et al (2010) Losartan prevents portal hypertension-induced, redox-mediated endothelial dysfunction in the mesenteric artery in rats. Gastroenterology 138: 1574–1584. 19879274 10.1053/j.gastro.2009.10.040

[12] Rabiller A, Nunes H, Lebrec D, Tazi KA, Wartski M, et al (2002) Prevention of gram-negative translocation reduces the severity of hepatopulmonary syndrome. Am J Respir Crit Care Med 166: 514–517. 12186830 10.1164/rccm.200201-027OC

[13] Wiest R, Garcia-Tsao G (2005) Bacterial translocation (BT) in cirrhosis. Hepatology 41: 422–433. 15723320 10.1002/hep.20632

[14] Wiest R, Das S, Cadelina G, Garcia-Tsao G, Milstien S, et al (1999) Bacterial translocation in cirrhotic rats stimulates eNOS-derived NO production and impairs mesenteric vascular contractility. J Clin Invest 104: 1223–1233. 10545521 10.1172/JCI7458 PMC409820

[15] Runyon BA, Borzio M, Young S, Squier SU, Guarner C, et al (1995) Effect of selective bowel decontamination with norfloxacin on spontaneous bacterial peritonitis, translocation, and survival in an animal model of cirrhosis. Hepatology 21: 1719–1724. 7768517

[16] Liu L, Liu N, Zhao Z, Liu J, Feng Y, et al (2012) TNF-alpha neutralization improves experimental hepatopulmonary syndrome in rats. Liver Int 32: 1018–1026. 22672643 10.1111/j.1478-3231.2012.02821.x

[17] Rasaratnam B, Kaye D, Jennings G, Dudley F, Chin-Dusting J (2003) The effect of selective intestinal decontamination on the hyperdynamic circulatory state in cirrhosis. A randomized trial. Ann Intern Med 139: 186–193. 12899586 10.7326/0003-4819-139-3-200308050-00008

[18] Tanikella R, Philips GM, Faulk DK, Kawut SM, Fallon MB (2008) Pilot study of pentoxifylline in hepatopulmonary syndrome. Liver Transpl 14: 1199–1203. 18668653 10.1002/lt.21482 PMC2834782

[19] Gupta N, Kumar A, Sharma P, Garg V, Sharma BC, et al.. (2013) Effects of the adjunctive probiotic VSL#3 on portal haemodynamics in patients with cirrhosis and large varices: a randomized trial. Liver Int. 10.1111/liv.12172 23601333

[20] Loguercio C, Federico A, Tuccillo C, Terracciano F, D'Auria MV, et al (2005) Beneficial effects of a probiotic VSL#3 on parameters of liver dysfunction in chronic liver diseases. J Clin Gastroenterol 39: 540–543. 15942443 10.1097/01.mcg.0000165671.25272.0f

[21] Sarr M, Chataigneau M, Martins S, Schott C, El Bedoui J, et al (2006) Red wine polyphenols prevent angiotensin II-induced hypertension and endothelial dysfunction in rats: role of NADPH oxidase. Cardiovasc Res 71: 794–802. 16822492 10.1016/j.cardiores.2006.05.022

[22] Chabot F, Mestiri H, Sabry S, Dall'Ava-Santucci J, Lockhart A, et al (1996) Role of NO in the pulmonary artery hyporeactivity to phenylephrine in experimental biliary cirrhosis. Eur Respir J 9: 560–564. 8730020 10.1183/09031936.96.09030560

[23] Barriere E, Tazi KA, Rona JP, Pessione F, Heller J, et al (2000) Evidence for an endothelium-derived hyperpolarizing factor in the superior mesenteric artery from rats with cirrhosis. Hepatology 32: 935–941. 11050042 10.1053/jhep.2000.19290

[24] Idris Khodja N, Chataigneau T, Auger C, Schini-Kerth VB (2012) Grape-derived polyphenols improve aging-related endothelial dysfunction in rat mesenteric artery: role of oxidative stress and the angiotensin system. PLoS One 7: e32039. 22384133 10.1371/journal.pone.0032039 PMC3288061

[25] Xavier FE, Aras-Lopez R, Arroyo-Villa I, Campo LD, Salaices M, et al (2008) Aldosterone induces endothelial dysfunction in resistance arteries from normotensive and hypertensive rats by increasing thromboxane A2 and prostacyclin. Br J Pharmacol 154: 1225–1235. 18500359 10.1038/bjp.2008.200 PMC2483383

[26] Criado M, Flores O, Vazquez MJ, Esteller A (2000) Role of prostanoids and nitric oxide inhibition in rats with experimental hepatic fibrosis. J Physiol Biochem 56: 181–188. 11198154 10.1007/BF03179785

[27] Kuhbacher T, Ott SJ, Helwig U, Mimura T, Rizzello F, et al (2006) Bacterial and fungal microbiota in relation to probiotic therapy (VSL#3) in pouchitis. Gut 55: 833–841. 16401690 10.1136/gut.2005.078303 PMC1856240

[28] Rehman A, Heinsen FA, Koenen ME, Venema K, Knecht H, et al (2012) Effects of probiotics and antibiotics on the intestinal homeostasis in a computer controlled model of the large intestine. BMC Microbiol 12: 47. 22452835 10.1186/1471-2180-12-47 PMC3338381

[29] Uronis JM, Arthur JC, Keku T, Fodor A, Carroll IM, et al (2011) Gut microbial diversity is reduced by the probiotic VSL#3 and correlates with decreased TNBS-induced colitis. Inflamm Bowel Dis 17: 289–297. 20564535 10.1002/ibd.21366 PMC2953593

[30] Chiva M, Soriano G, Rochat I, Peralta C, Rochat F, et al (2002) Effect of Lactobacillus johnsonii La1 and antioxidants on intestinal flora and bacterial translocation in rats with experimental cirrhosis. J Hepatol 37: 456–462. 12217598 10.1016/s0168-8278(02)00142-3

[31] Pardo A, Bartoli R, Lorenzo-Zuniga V, Planas R, Vinado B, et al (2000) Effect of cisapride on intestinal bacterial overgrowth and bacterial translocation in cirrhosis. Hepatology 31: 858–863. 10733540 10.1053/he.2000.5746

[32] Gobbetti M, Ferranti P, Smacchi E, Goffredi F, Addeo F (2000) Production of angiotensin-I-converting-enzyme-inhibitory peptides in fermented milks started by Lactobacillus delbrueckii subsp. bulgaricus SS1 and Lactococcus lactis subsp. cremoris FT4. Appl Environ Microbiol 66: 3898–3904. 10966406 10.1128/aem.66.9.3898-3904.2000 PMC92236

[33] Minervini F, Algaron F, Rizzello CG, Fox PF, Monnet V, et al (2003) Angiotensin I-converting-enzyme-inhibitory and antibacterial peptides from Lactobacillus helveticus PR4 proteinase-hydrolyzed caseins of milk from six species. Appl Environ Microbiol 69: 5297–5305. 12957917 10.1128/AEM.69.9.5297-5305.2003 PMC194939

[34] Sipola M, Finckenberg P, Santisteban J, Korpela R, Vapaatalo H, et al (2001) Long-term intake of milk peptides attenuates development of hypertension in spontaneously hypertensive rats. J Physiol Pharmacol 52: 745–754. 11785770

[35] Hata Y, Yamamoto M, Ohni M, Nakajima K, Nakamura Y, et al (1996) A placebo-controlled study of the effect of sour milk on blood pressure in hypertensive subjects. Am J Clin Nutr 64: 767–771. 8901799 10.1093/ajcn/64.5.767

[36] Rajagopalan S, Kurz S, Munzel T, Tarpey M, Freeman BA, et al (1996) Angiotensin II-mediated hypertension in the rat increases vascular superoxide production via membrane NADH/NADPH oxidase activation. Contribution to alterations of vasomotor tone. J Clin Invest 97: 1916–1923. 8621776 10.1172/JCI118623 PMC507261

