# Correction: Probiotics (VSL#3) Prevent Endothelial Dysfunction in Rats with Portal Hypertension: Role of the Angiotensin System

**DOI:** 10.1371/journal.pone.0106467

**Published:** 2014-08-19

**Authors:** The PLOS ONE Staff

The second author’s name is spelled incorrectly. The correct name is: Noureddine Idris-Khodja. The correct citation is: Rashid SK, Idris-Khodja N, Auger C, Alhosin M, Boehm N, et al. (2014) Probiotics (VSL#3) Prevent Endothelial Dysfunction in Rats with Portal Hypertension: Role of the Angiotensin System. PLoS ONE 9(5): e97458. doi:10.1371/journal.pone.0097458
